# Nanotherapeutics Containing Lithocholic Acid-Based Amphiphilic Scorpion-Like Macromolecules Reduce In Vitro Inflammation in Macrophages: Implications for Atherosclerosis

**DOI:** 10.3390/nano8020084

**Published:** 2018-02-02

**Authors:** Alysha Moretti, Qi Li, Rebecca Chmielowski, Laurie B. Joseph, Prabhas V. Moghe, Kathryn E. Uhrich

**Affiliations:** 1 Department of Chemistry and Chemical Biology, Rutgers University, Piscataway, NJ 08854, USA; aemoretti425@gmail.com; 2 Department of Biomedical Engineering, Rutgers University, Piscataway, NJ 08854, USA; atheoi17@gmail.com (Q.L.); moghe@soe.rutgers.edu (P.V.M.); 3 Department of Chemical and Biochemical Engineering, Rutgers University, Piscataway, NJ 08854, USA; chmielow@scarletmail.rutgers.edu; 4 Department of Pharmacology and Toxicology, Rutgers University, Piscataway, NJ 08854, USA; lbjoseph@pharmacy.rutgers.edu; 5 Department of Chemistry, University of California-Riverside, Riverside, CA 92521, USA

**Keywords:** atherosclerosis, amphiphile, TGR5, lithocholic acid, nanoparticle, inflammation, macrophages

## Abstract

Previously-designed amphiphilic scorpion-like macromolecule (AScM) nanoparticles (NPs) showed elevated potency to counteract oxidized low-density lipoprotein (oxLDL) uptake in atherosclerotic macrophages, but failed to ameliorate oxLDL-induced inflammation. We designed a new class of composite AScMs incorporating lithocholic acid (LCA), a natural agonist for the TGR5 receptor that is known to counteract atherosclerotic inflammation, with two complementary goals: to simultaneously decrease lipid uptake and inhibit pro-inflammatory cytokine secretion by macrophages. LCA was conjugated to AScMs for favorable interaction with TGR5 and was also hydrophobically modified to enable encapsulation in the core of AScM-based NPs. Conjugates were formulated into negatively charged NPs with different core/shell combinations, inspired by the negative charge on oxLDL to enable competitive interaction with scavenger receptors (SRs). NPs with LCA-containing shells exhibited reduced sizes, and all NPs lowered oxLDL uptake to <30% of untreated, human derived macrophages in vitro, while slightly downregulating SR expression. Pro-inflammatory cytokine expression, including IL-1β, IL-8, and IL-10, is known to be modulated by TGR5, and was dependent on NP composition, with LCA-modified cores downregulating inflammation. Our studies indicate that LCA-conjugated AScM NPs offer a unique approach to minimize atherogenesis and counteract inflammation.

## 1. Introduction

Cardiovascular disease is the leading global cause of death, claiming approximately 17.3 million lives and costing upwards of $860 billion annually [1]. Innovative methods for prevention and treatment of cardiovascular disease are needed to decrease its prevalence. Many vascular conditions begin with atherosclerosis, an inflammatory cascade culminating in the accumulation of atherosclerotic lesions and calcified plaques in the arterial vasculature [2]. In atherogenesis, circulating monocytes are recruited in response to subendothelial retention of apolipoprotein B and oxidative modification of low density lipoproteins (oxLDL). Macrophages, differentiated from strongly adherent monocytes, become lipid laden foam cells as they internalize oxLDL via two primary scavenger receptors (SRs), SR-A and CD36 [3,4]. Excessive and unregulated oxLDL internalization ultimately results in macrophage apoptosis, and ineffective clearance of cellular debris initiates plaque formation around a necrotic core leading to adverse cardiovascular events [4]. 

Statins represent the most commonly prescribed class of cholesterol-lowering pharmaceuticals. Statins function via competitive inhibition of HMG-CoA reductase, the rate-limiting step of cholesterol biosynthesis, and subsequently upregulate low density lipoprotein (LDL) receptors [5,6]. Although they exhibit substantially decreased hepatic and circulating cholesterol levels, systemic administration and off-target effects prove detrimental [7,8]. HMG-CoA reductase inhibition also affects synthesis of natural products synthesized downstream of cholesterol (e.g., bile acids, sex-steroids), as well as crucial parallel biosynthetic pathways (e.g., coenzyme Q10) [8]. As such, strategically targeted approaches to ameliorate atherosclerosis are needed, including techniques to lower the recruitment of monocytes, inhibit the deposition and accumulation of circulating LDL and atherogenic lipids at lesion sites, and counteract the athero-inflammatory cascade that leads to lesion growth. 

**Figure 1:**
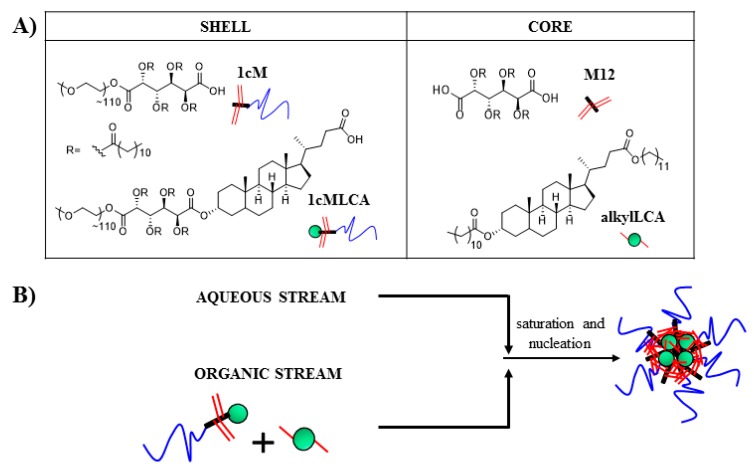
Chemical structures and schematics of shell and core molecules incorporated into NPs via flash nanoprecipitation. Chemical structures of 1cM and 1cMLCA shells and M12 and alkylLCA cores and corresponding cartoons (**A**). R group refers to dodecyl ester (top left) in all chemical structures. Schematic representation of the flash nanoprecipitation technique utilized to generate amphiphilic scorpion-like macromolecule (AScM) nanoparticles (NPs) using 1cMLCA[alkylLCA] as an example (**B**) where the poly(ethylene glycol) (PEG) components (blue lines) extend into aqueous media from the shell and lithocholic acid (LCA) (green circles) is encapsulated within the core of the NP. Nomenclature of 1cM refers to 1 carboxylic acid (1c) on a mucic acid backbone (M), and M12 nomenclature refers to a mucic acid backbone (M) with dodecyl alkyl chains (12).

Amphiphilic scorpion-like macromolecules (AScMs) designed in our group have been shown to attenuate atherosclerotic outcomes [9,10,11,12,13,14]. AScMs are comprised of a linear sugar backbone with fatty acid pendants conjugated to poly(ethylene glycol) (PEG), which self-assemble into nanoscale micelles at low concentrations (~10^−6^ M). These unique compounds can successfully encapsulate and deliver anti-atherosclerotic therapeutics to macrophages, increasing drug bioavailability [11,15]. Furthermore, the AScMs exhibit inherent bioactivity against several cell types (e.g., macrophages and smooth muscle cells) involved in the atherosclerotic cascade when formulated into either micelles or kinetically trapped nanoparticles (NPs), which are advantageous due to their resistance to dissociation upon dilution [16,17,18]. NPs have demonstrated additional superiority to micelle formulations as AScM NPs lower macrophage oxLDL uptake to ~25% relative to untreated controls, decrease SR expression, and reduce plaque size and aortic occlusion in vivo [17,19,20]. AScMs mode of action occurs via competitive inhibition of oxLDL uptake by SRs due to columbic interactions between the negative charge on NPs and the positive charge in SR binding pockets [13]. Investigation into interactions between differentially oxidized LDL and AScM NPs indicate negligible interactions and support this primary mode of action [10]. Despite their promise for treating early stage atherosclerosis, the lead NP formulation, consisting of a 1cM shell and M12 core (Figure 1), fails to ameliorate the inflammatory component of atherosclerosis that can exacerbate the disease state [18]. Thus, new AScM designs are necessary to not only counteract atherogenesis, but also mitigate the accompanying inflammatory cascade. 

Recent approaches to mitigate this effect include using anti-inflammatory molecules, such as Vitamin E, as the NP core component; however, these NPs exhibit compromised oxLDL uptake inhibition at low administration concentrations, and this strategy does not target the intrinsic inflammatory responses accompanying oxidized lipid uptake [18]. An alternate preventative approach is to target inherent biological lipogenic pathways that combat the inflammatory response induced by both the NPs and the atherosclerotic cascade. The G-protein coupled bile acid receptor 1 (GPBAR-1, also known as TGR5), known for its anti-inflammatory implications in the digestive tract, is an emerging target in mediating pro-inflammatory cytokines in atherosclerosis due to its high expression in both monocytes and macrophages [21]. Activation of TGR5 reportedly reduces monocyte adhesion in endothelial cells, stabilizes the alternative M2 phenotype, and inhibits lesion formation in vivo [21,22,23,24]. Lithocholic acid (LCA) is a naturally occurring secondary bile acid resulting from the dehydroxylation of chenodeoxycholic acid, and activates TGR5 at 0.53 µM [21]. LCA, which is present in serum in its free and conjugated forms, has the strongest activation of TGR5 compared to other naturally occurring bile acids such as deoxycholic acid, chenodeoxycholic acid, and cholic acid. Furthermore, LCA has demonstrated suppressed lipopolysaccharide (LPS)-stimulated cytokine production and has anti-atherogenic implications [25]. Hence, conjugation of LCA to AScMs could decrease the inherent inflammation associated with AScM NP administration and provide benefits of activating a target implicated in anti-atherogenic outcomes both in vitro and in vivo*.*

Here, we report the synthesis of LCA-conjugated NP components and describe their bioactivity in human macrophages in vitro. LCA-conjugated constructs were strategically designed based on the hypothesis that native esterases would cleave AScMs and act as depots that release LCA to interact with TGR5 without compromising the anti-atherogenic effects of the AScM NPs. NP formulations with unique core/shell combinations were evaluated for the components’ influence on NP physicochemical properties, as well as the ability to inhibit oxLDL uptake and lower macrophage inflammation.

## 2. Materials and Methods

### 2.1. Materials

All reagents and materials were purchased from Sigma-Aldrich (Milwaukee, WI, USA) and were used as received unless noted otherwise. Hydrochloric acid (HCl, 1 N) was purchased from Fisher Scientific (Fair Lawn, NJ, USA), and silica from VWR (Radnor, PA, USA). The confined impinging jet mixer was provided by Prof. Robert Prud’homme at Princeton University (Princeton, NJ, USA). Cell culture assays used human buffy coats purchased from the New York Blood Center (New York, NY, USA) or New Jersey Blood Center (East Orange, NJ, USA), Ficoll-Paque premium 1.077 g/mL and Percoll 1.3 g/mL from GE Healthcare (Fairfield, CT, USA), FEP Teflon cell culture bags from Cellgenix, RPMI-1640 from ATCC (Manassas, VA, USA), macrophage colony stimulating factor (MCSF) from PeproTech (Rocky Hill, NJ, USA), penicillin/streptomycin from Lonza (Basel, Switzerland), fetal bovine serum (FBS) from Life Technologies (Carlsbad, CA, USA), human serum from MP Biomedical, unlabeled oxLDL from Biomedical Technologies Inc. (Ward Hill, MA, USA), and 3,3′-dioctadecyloxacarbocyanine (DiO) labeled oxLDL from Kalen Biomedical (Montgomery Village, MD, USA). 

### 2.2. Characterization

Proton (^1^H) and carbon (^13^C) nuclear magnetic resonance (NMR) spectra were obtained from a Varian 400 MHz or 500 MHz spectrometer. Reaction products were dissolved in deuterated chloroform (CDCl_3_) with trimethylsilane (TMS) as an internal reference. Fourier transform infrared (FT-IR) spectra were obtained by solvent-casting small molecules onto sodium chloride (NaCl) plates in dichloromethane (DCM) then recorded using a Thermo Scientific Nicolet iS10 spectrophotometer with an average of 32 scans per sample and processed using OMNIC software. Small molecule molecular weights were established using a ThermoQuest Finnigan (LCQ-DUO system equipped with a syringe pump, optional divert/inject valve, atmospheric pressure ionization (API) source, and mass spectrometer (MS) detector) and spectra were processed using the Xcalibur data system. Compounds (10 µg/mL) were dissolved in methanol (MeOH) or DCM with 1% acetic acid or ammonia for positive or negative ion detection, respectively. Weight-averaged molecular weights (Mw) and polydispersity indices (PDI) of AScMs were determined by gel permeation chromatography (GPC) using a Waters liquid chromatography (LC) system (Milford, MA, USA), equipped with a 2414 refractive index detector, 1515 isocratic HPLC pump, 717plus autosampler, and Jordi divinylbenzene mixed-bed GPC column (7.8 × 300 mm, Alltech Associates, Deerfield, IL, USA). Samples (10 mg/mL) were prepared in DCM and filtered through a 0.45 µm polytetrafluoroethylene (PTFE) syringe filter prior to autoinjection. The eluent (DCM) was set at a flow rate of 1 mL/min, and IBM ThinkCentre computer with WaterBreeze version 3.20 software used to process data against a calibration curve generated with broad-range PEG standards (Waters Milford, MA, USA). 

### 2.3. Synthesis

#### 2.3.1. Synthesis of 1cMLCA (**4**)

According to a modified literature procedure, the carboxylic acid of LCA was selectively protected [26]. LCA (**1**, 1.0 equivalents (eq.)) was dissolved in 10 mL of anhydrous dimethylformamide (DMF) under nitrogen. Potassium carbonate (1.2 eq.) was added and the suspension allowed to stir for 30 min. Benzyl bromide (1.5 eq.) was then added and the reaction stirred for 12 h at room temperature. The reaction was diluted with diethyl ether (20 mL) and washed with sodium bicarbonate (3×, 15 mL). The crude product was dried over magnesium sulfate (MgSO_4_), filtered, and concentrated in vacuo. **2** was then purified on silica gel via flash chromatography using a hexanes/ethyl acetate gradient (95:5 to 80:20). 

1cM, synthesized as previously described [27], was dried by azeotropic distillation with toluene under reduced pressure 3× prior to use. 1cM (1.0 eq.) was then dissolved in 10 mL of DCM under nitrogen with **2** (2.0 eq.) and 4-(dimethylamino)pyridinium-4-toluene sulfonate (DPTS, 1.0 eq.) under nitrogen, followed by dropwise addition of *N*,*N*′-dicyclohexylcarbodiimide (DCC, 1 M, 2.0 eq.) as a coupling reagent. After 24 h, the reaction was cooled to 0 °C and the urea byproduct removed via filtration. The filtrate was washed 2× with 1 N HCl and 1× with brine, followed by removal of solvent in vacuo. The product was precipitated in diethyl ether (50 mL), collected via centrifugation (3500 rpm, 5 min each) and washed a total of 5× to obtain **3**. 

The benzyl group was removed via hydrogenolysis with palladium on carbon (Pd/C, 10% *w*/*w*) as the catalyst for 24 h in DCM (10 mL). The heterogeneous mixture was then filtered through Celite to remove Pd/C and the filtrate removed in vacuo to obtain **4**. 

*Benzyl lithocholate* (**2**): Yield: 76% (white solid). ^1^H-NMR (500 MHz, CDCl_3_): δ 0.61 (s, 3H), 0.91 (s, 6H), 0.94–1.93 (br. m, 27H), 2.26 (m, 1H), 2.39 (m, 1H), 3.61 (m, 1H), 5.10 (s, 2H), 7.35 (m, 5H). ^13^C-NMR (500 MHz, CDCl_3_): δ 12.01, 18.23, 20.80, 23.36, 24.19, 26.40, 27.18, 28.16, 30.54, 30.96, 31.27, 34.56, 35.30, 35.34, 35.83, 36.45, 40.15, 40.41, 42.08, 42.71, 55.94, 56.47, 66.07, 71.85, 128.15, 128.21, 128.53, 136.12, 174.11. IR (cm^−1^, thin film from chloroform CHCl_3_): 3334.36 (OH, alcohol), 1736.88 (C=O, ester). Electrospray ionization-mass spectrometry (ESI-MS) *m*/*z*: 505.5 [M + 39]^+^.

*1cM benzyl lithocholate* (**3**): Yield: 78% (white solid) ^1^H-NMR (500MHz, CDCl_3_): δ 0.64 (s, 3H), 0.87 (m, 18H), 0.94–1.99 (br. m. 99H), 2.27 (m, 4H), δ 2.41 (m, 4H), 3.63 (m, ~425H), 4.27 (m, 2H), 4.30 (m, 2H), 4.72 (m, 1H), 5.18 (m, 4H), 5.70 (m, 2H), 7.35 (m, 5H). *M*w = 5.6, PDI = 1.2.

*1cMLCA* (**4**): Yield: 81% (white solid) ^1^H-NMR (500MHz, CDCl_3_): δ 0.64 (s, 3H), 0.87 (m, 21H), 0.94–1.99 (br. m. 99H), 2.27 (m, 4H), 2.41 (m, 4H), 3.63 (m, ~425H), 4.27 (m, 2H), 4.30 (m, 2H), 4.72 (m, 1H), 5.18 (m, 2H), 5.70 (m, 2H). *M*w = 5.6, PDI = 1.2.

#### 2.3.2. Synthesis of LCA-Based Hydrophobe (**7**)

**2** (1.0 eq.) and zinc chloride (ZnCl_2_, 0.5 eq.) were dissolved in 10 mL of DCM under N_2_, followed by addition of lauroyl chloride (3.0 eq.), then heated to reflux overnight with stirring. The crude product was then purified using silica gel via flash chromatography with hexanes/ethyl acetate (90:10) to yield **5**. 

The benzyl-protecting group was removed via hydrogenolysis as previously described, and the carboxylic acid of **6** was conjugated to dodecanol via carbodiimide coupling. Dodecanol (2.0 eq.) and dimethylaminopyridine (DMAP, 2.0 eq.) were completely dissolved in 10 mL anhydrous DCM under nitrogen. The coupling reagent 1-ethyl-3-(3-(dimethylamino)propyl) carbodiimide (EDCI, 2.5 eq.) was added and the reaction stirred overnight. The mixture was washed with 10% potassium bisulfite (2×, 15 mL) and brine (1×, 15 mL) to remove the urea byproduct and DMAP. The crude product was purified on silica gel via flash chromatography with hexanes/ethyl acetate (80:20). The organic layer was then dried over MgSO_4_, filtered, and concentrated in vacuo to obtain **7**. 

*Alkylated benzyl lithocholate* (**5**): Yield: 73% (white solid). ^1^H-NMR (500 MHz, CDCl_3_): δ 0.61 (s, 3H), 0.88 (t, 3H), 0.91(s, 6H), 0.94–1.93 (br. m, 44H), 2.26 (m, 3H), 2.39 (m, 1H), 4.72 (m, 1H), 5.10 (s, 2H), 7.35 (m, 5H). ^13^C-NMR (500 MHz, CDCl_3_): δ 12.01, 14.10, 18.24, 20.82, 22.67, 23.32, 24.16, 25.09, 26.32, 26.69, 27.02, 28.16, 29.12, 29.24, 29.32, 29.45, 29.59, 30.97, 31.28, 31.90, 32.27, 34.59, 34.78, 35.04, 35.31, 35.78, 40.12, 40.39, 41.90, 42.72, 56.00, 56.46, 66.07, 74.05, 128.15, 128.21, 128.52, 136.12, 173.43, 174.08. IR (cm^−1^, thin film from chloroform CHCl_3_): 1735.05 (C=O, ester). 

*Mono-alkylated lithocholic acid* (**6**): Yield: quantitative (white solid) ^1^H-NMR (500 MHz, CDCl_3_): δ 0.64 (s, 3H), 0.88 (t, 3H), 0.92 (s, 6H), 0.94–1.96 (br. m, 44H), 2.26 (m, 3H), 2.39 (m, 1H), 4.72 (m, 1H). ^13^C-NMR (500 MHz, CDCl_3_): δ 12.03, 14.10, 18.23, 20.82, 22.67, 23.32, 24.16, 25.09, 26.31, 26.68, 27.01, 28.16, 29.11, 29.24, 29.32, 29.45, 29.58, 29.59, 30.75, 30.82, 31.90, 32.29, 34.58, 34.78, 35.04, 35.30, 35.78, 40.13, 30.39, 41.89, 42.74, 55.97, 56.47, 74.06, 173.48, 179.36. IR (cm^−1^, thin film from chloroform CHCl_3_): 1732.86 (C=O, ester), 1703.15 (C=O, acid). ESI-MS *m*/*z*: 557.7 [M − 1]^−^.

*Alkylated lithocholate* (**7**): Yield: 86% (white solid) ^1^H-NMR (500 MHz, CDCl_3_): δ 0.64 (s, 3H), 0.87 (t, 3H), 0.92–1.96 (br. m, 71H), 2.30 (m, 6H), 4.04 (t, 2H), 4.77 (m, 1H). ^13^C-NMR (500 MHz, CDCl_3_): δ 12.02, 14.10, 18.25, 20.82, 22.67, 23.32, 24.17, 25.09, 25.94, 26.34, 26.68, 27.02, 28.17, 28.65, 29.11, 29.24, 29.32, 29.34, 29.45, 29.52, 29.56, 29.58, 29.59, 29.62, 29.64, 31.05, 31.36, 31.91, 32.29, 34.59, 34.78, 35.04, 35.34, 35.79, 40.13, 40.39, 41.90, 42.73, 56.04, 56.47, 64.42, 74.05, 173.44, 174.42. IR (cm^−1^, thin film from chloroform CHCl_3_): 1737.47 (C=O, ester). ESI-MS *m*/*z*: 727.5 [M + 1]^+^.

### 2.4. Nanoparticle Fabrication

NPs were fabricated via flash nanoprecipitation as previously described [17]. Briefly, the AScM (40 mg/mL) and hydrophobe (20 mg/mL) were separately dissolved in tetrahydrafuran (THF). A 1:1 *v*/*v* mixture of the AScM:hydrophobe solution (0.5 mL) was filtered through a 0.2 µm PTFE filter, then rapidly mixed with phosphate buffered saline (PBS, 0.5 mL) in a confined impinging jet mixer, and subsequently added to 4.5 mL of PBS. NP suspensions were dialyzed using a 6–8 kDa ultrafiltration membrane cut-off 3× against sterile PBS (2 L) for organic solvent removal.

### 2.5. Nanoparticle Characterization

NP sizes and zeta (ζ) potential were measured by dynamic light scattering (DLS) using a Malvern-Zetasizer Nano Series (ZS90) in triplicate with a 90° scattering angle. NPs sizes and PDI were evaluated in PBS, and the Z-average was taken as the hydrodynamic diameter. Prior to analyzing ζ potential, NPs were dialyzed extensively against deionized water.

### 2.6. Isolation of Human Monocyte Derived Macrophages (hMDMs)

Peripheral blood mononuclear cells (PBMCs) were isolated from human buffy coats by centrifugation through Ficoll-Paque (1.077 g/cm^3^) density gradient [19]. Red blood cells were lysed with ammonium-chloride-potassium (ACK) buffer and cell debris, including platelets, were removed via centrifugation (300× *g*, 10 min). PBMCs were washed with PBS and cultured in RPMI 1640 supplemented with 10% fetal bovine serum (FBS) and 1% penicillin/streptomycin (complete medium). PBMCs were selected for monocytes as determined by flask adherence after 24 h at 37 °C and 5% CO_2_. Monocytes were cultured for 7 days in complete medium with 50 ng/mL macrophage colony stimulating factor (M-CSF) to differentiate monocytes into macrophages. hMDMs were trypsinized, and plated at a density of 150,000 cells/mL and let rest for a minimum of 12 h prior to experimentation.

### 2.7. Oxidized Low Density Lipoprotein (oxLDL) Uptake in Macrophages

hMDMs were plated in 24 well tissue culture plates at a density of 1.5 × 10^5^ cells/mL and incubated with unlabeled oxLDL (4 µg/mL) and DiO labeled oxLDL (1 µg/mL) in the presence of NPs (10^−5^ M) in complete medium for 24 h, as this concentration was demonstrated to have >80% cell viability. Treatment and control media were aspirated and replaced with ice-cold PBS containing 2 mM ethylenediaminetetraacetic acid (EDTA) and plates were placed on ice packs. Cells and EDTA were triturated to remove cells from plates, centrifuged (1000 rpm, 10 min), and fixed in 1% paraformaldehyde (150 µL). oxLDL uptake was quantified by fluorescence using a FACScalibur flow cytometer (Becton Dickenson, Franklin Lakes, NJ, USA) by collecting a minimum of 10,000 events/sample. Results were analyzed via FlowJo software (Tree Star Inc., Ashland, OR, USA) and reported as the geometric mean fluorescence intensity (MFI). All experiments were performed in triplicate and data is presented as percent (%) oxLDL uptake as normalized to the basal control. 

### 2.8. Gene Expression in Macrophages

Gene expression (GAPDH, ACTB, IL-1β, IL-6, IL-8, IL-10, TNFα, CD36, SR-A) in hMDMs was evaluated using quantitative reverse transcription polymerase chain reaction (qRT-PCR). RNA was extracted from hMDMs 24 h after treatment using an RNeasy Plus Mini Kit with Quiashredder columns according to supplier protocol (Qiagen). The concentration and purity of RNA was quantified using a Nanodrop 2000c. RNA was reverse transcribed to cDNA using a High Capacity cDNA Kit and RapidCycler thermal cycler (Idaho Technology). RT-PCR was carried out using a Lightcycler 480 (Roche) with Fast SYBR Green Master Mix for 45 cycles. Fold-change was calculated using ΔΔCt method and normalized to housekeeping genes (actin-β and GAPDH). All forward and reverse primers were designed by Harvard Primer Bank or Primer-BLAST and synthesized by Integrated DNA Technology. 

### 2.9. Confocal Microscopy of oxLDL Uptake in Macrophages

Uptake of oxLDL was visualized using confocal microscopy. PBMCs were isolated from human buffy coats by Ficoll-Paque (1.077 g/cm^3^) density gradient and Percoll (1.131 g/cm^3^) density gradient. PBMCs were collected and washed with PBS-ETDA (1 mM) and plated into FEP Teflon-coated cell culture bags at a density of at least 5.0 × 10^7^ monocytes per bag [28]. Monocytes were differentiated into M2 macrophages using recombinant human M-CSF (2.5 ng/mL) and incubated at 37 °C in 5% CO_2_ in complete medium for 7 d. After 7 d, culture bags were placed on ice for at least 1 h. The cell suspension was removed using a syringe, and cells were isolated by centrifugation (400 *g*, 10 min). The cells were re-plated at a density of 150,000 cells/mL for at least 12 h prior to treatment. After 24 h treatment, cells were washed with PBS (pH 7.4, 3×) and fixed with 4% PFA for 20 min. The PFA was removed and Hoescht dye (0.1 µg/mL) was added for 15 min. Cells were imaged using a Leica TCS SP8 confocal microscope with a 40× oil immersion objective.

### 2.10. Statistical Analysis

Statistical analyses were performed using Graph Pad Prism V7.01. Statistical significance was determined using a one-way ANOVA with Tukey’s posthoc test for comparisons between multiple groups. Statistical significance of *p* ≤ 0.05 is indicated in figures. 

## 3. Results and Discussion

### 3.1. LCA-Conjugates Successfully Synthesized via Multi-Step Reactions

LCA-conjugates were synthesized to evaluate the effect of incorporating a naturally occurring TGR5 ligand into AScM NP formulations to address atherosclerotic inflammation and mitigate the proinflammatory effects observed with AScM NP administration. Both an LCA-conjugated AScM and a hydrophobically modified LCA-conjugate were synthesized to enable the fabrication of NPs with high LCA incorporation in the shell and core, respectively.

**Scheme 1:**
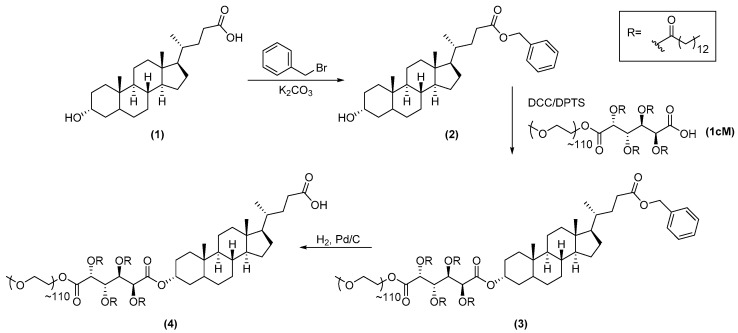
Synthetic approach for LCA-conjugated AScM.

LCA-conjugates were strategically designed to retain oxLDL uptake reduction properties, while exhibiting the active LCA functionality. We previously observed that AScMs with a net negative charge resulted in statistically significant reductions in oxLDL uptake compared to neutral or cationic analogs [9]. The 1cMLCA-conjugate was designed such that LCA would be at the terminal end to freely interact with TGR5 and bear a net negative charge to mimic the physicochemical properties of oxLDL for effective SR competitive inhibition and cellular uptake [29]. The carboxylic acid of LCA (**1**) was initially protected by reaction with benzyl bromide in the presence of a mild base as shown in Scheme 1. By doing so, it was assured that the lead AScM from previous studies (1cM) would be conjugated through the hydroxyl group of **2** via subsequent carbodiimide coupling, yielding the protected 1cMLCA-conjugate (**3**). The benzyl protecting group was then removed via hydrogenolysis with 10% *w*/*w* Pd/C to yield the final 1cMLCA-conjugate (**4**). 

**Scheme 2:**
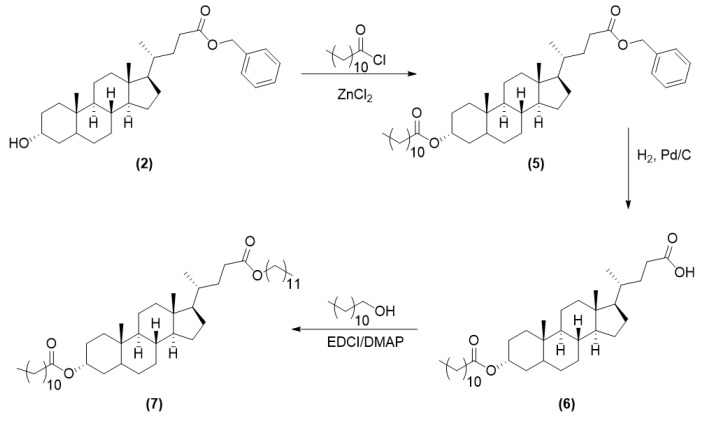
Synthetic approach for LCA-based hydrophobe.

A hydrophobic analog of LCA was also synthesized according to Scheme 2 to enable fabrication of NPs via flash nanoprecipitation. This method combines an amphiphile (e.g., 1cMLCA) and hydrophobe (e.g., alkylLCA) in a water-miscible organic solvent prior to fabrication. The organic solution is then rapidly mixed with an aqueous buffer, resulting in hydrophobe precipitation. The amphiphile assembles around the precipitates, generating NPs with a specific core/shell architecture (Figure 1B) [30,31]. Although LCA has low aqueous solubility and is capable of forming NPs with AScMs, the resulting NP stability proved to be insufficient, resulting in rapid NP aggregation (data not shown). Ansell et al. have previously demonstrated that increasing the hydrophobicity of partially water-soluble drugs via lipophilic anchor conjugation improves NP formation, extending the half-life, and thus, bioactivity [32]. To enable efficient encapsulation of LCA and improve NP stability, the lipophilicity of LCA was increased by first acylating the hydroxyl of **2** in the presence of zinc (II) chloride to yield **5**. Following benzyl deprotection via hydrogenolysis, the free carboxylic acid was alkylated via carbodiimide coupling to yield the LCA-based hydrophobe, alkylLCA (**7**) in high purity. 

**Figure 2:**
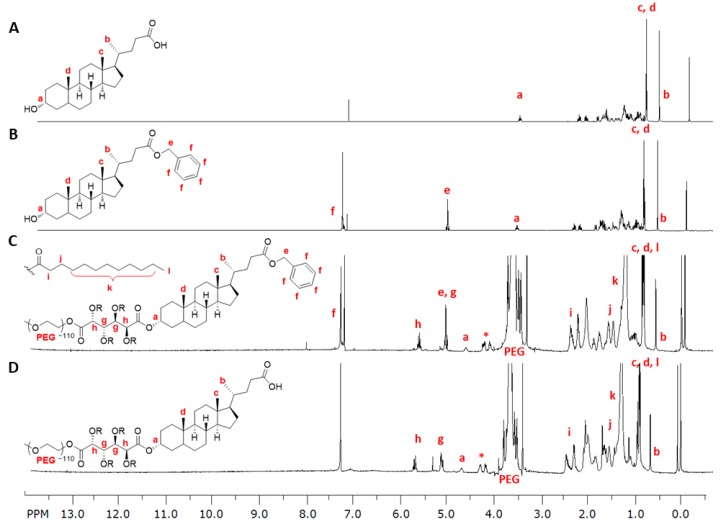
Sequential ^1^H-nuclear magnetic resonance (NMR) spectra in the synthesis of 1cMLCA. (**A**) LCA; (**B**) benzyl lithocholate; (**C**) 1cM benzyl lithocholate; (**D**) 1cMLCA. (*) represents methylene group in PEG adjacent to the ester.

All small molecules were characterized via ^1^H-NMR, ^13^C-NMR, and FT-IR spectroscopies and ESI-MS, while PEGylated products were characterized via ^1^H-NMR spectroscopy and GPC. Several techniques, including ^1^H-NMR and FT-IR spectroscopies were critical to elucidate the final structures of LCA-conjugates and precursors. Figure 2 presents the sequential ^1^H-NMR spectra leading to the synthesis of 1cMLCA. Successful benzyl protection of LCA’s acid functionality was confirmed via the absence of the acid carbonyl and the appearance of an ester stretch at 1736.33 cm^−1^ in the FT-IR spectrum. Additionally, benzylic and aromatic protons at 5.10 ppm and 7.35 ppm, respectively, in the ^1^H-NMR spectrum were indicative of successful protection (e and f in Figure 2). Conjugation of **2** to 1cM to generate **3** was evidenced by the relative integration of peaks from **2** and those previously established to be characteristic of 1cM, as well as the downfield chemical shift of the C3 hydrogen from 3.61 ppm to 4.72 ppm (a in Figure 2). Generation of the final 1cMLCA conjugate (**4**) was confirmed via the disappearance of aromatic and benzylic protons in the ^1^H-NMR spectra. 

**Figure 3:**
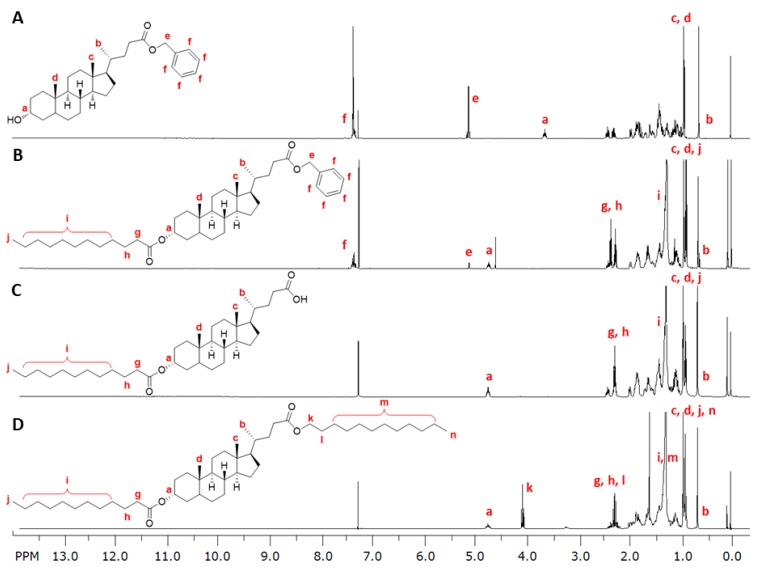
Sequential ^1^H-NMR spectra in the synthesis of LCA-based hydrophobe. (**A**) benzyl lithocholate; (**B**) alkylated benzyl lithocholate; (**C**) monoalkylated LCA; (**D**) alkylated lithocholate.

Techniques including ^1^H-NMR and FT-IR spectroscopies, and ESI-MS were also crucial in confirming successful generation of alkylLCA (**7**). A downfield shift of the C3 hydrogen from 3.61 ppm to 4.72 ppm and disappearance of the broad –OH stretching vibration in the FT-IR spectrum confirmed hydroxyl group acylation. Benzyl deprotection was evidenced by the disappearance of benzylic and aromatic protons (e and f in Figure 3) as previously described, as well as a parent peak in the ESI-MS at *m*/*z* = 557.7 [M − 1]^−^. Subsequent alkylation of the carboxylic acid was confirmed via the appearance of a triplet representing the methylene adjacent to the ester at 4.04 ppm (k in Figure 3) and disappearance of the acid carbonyl in the FT-IR spectra at 1703.15 cm^−1^.

### 3.2. Stable Mono-Dispersed Nanoparticles Fabricated with LCA-Conjugates Possess Negative Charge and Desirable Size Distributions

Although previous AScM micellar preparations were shown to have bioactivity at high concentrations, improved inhibition of oxLDL uptake was observed with AScM NPs in serum-containing medium compared to micelles [16,17]. NPs fabricated using flash nanoprecipitation are different from micelles in that the core molecule serves as a nucleation point to fabricate kinetically trapped particles that do not dissociate upon dissolution. [17] In the present study, novel AScM NPs were generated via flash nanoprecipitation, including 1cMLCA and alkylLCA as the shell and core components, respectively. Additional NP formulations with combinations of shell and core materials were also prepared to evaluate the potential impact of each component on physicochemical properties and biological outcomes. The previously demonstrated highly efficacious core (i.e., M12) and shell (i.e., 1cM) materials were included in the evaluation for comparison (Figure 1A). 

The physicochemical properties of NP formulations are shown in Table 1. The data demonstrates that shell composition influences particle size, while core chemistry does not have a significant influence on any physical attributes. NPs prepared with 1cM shells were found to have statistically indistinct hydrodynamic diameters, regardless of core identity. Likewise, NPs with 1cMLCA shells have similar sizes, independent of the core material. However, NPs prepared with 1cM shells have hydrodynamic diameters that are statistically larger than NPs made with 1cMLCA shells (*p* ≤ 0.05). The discrepancy may be due to the differential dynamics of various components during the flash nanoprecipitation process. This observation suggests that the core materials have similar rates of precipitation when the molecules contact the aqueous stream during flash nanoprecipitation. However, the aggregation and packing behavior of the shell material around the precipitated cores appears to differ due to the addition of the LCA moiety. It is possible that the increased hydrophobicity of LCA also enhances the packing density of AScMs around the core, therefore leading to the decreased hydrodynamic diameters of NPs with 1cMLCA shells.

Interestingly, NPs with 1cMLCA shells have sizes below 200 nm, which is the generally accepted upper limit for cellular uptake via endocytosis [33]. Previous data demonstrated a direct correlation between AScM NP internalization by cells and an increase in oxLDL uptake inhibition efficacy, and smaller particles have been shown to be more effectively internalized via endocytosis [17,20,33]. Further, all NPs have comparable negative ζ potentials, an attribute which appears crucial to NP efficacy for interactions with cationic SRs. This result correlates with previous data, as NPs with a negative ζ potential are hypothesized to allow AScM NPs to competitively inhibit oxLDL uptake via coulombic interactions with the positive charge in SR binding pockets [10,29]. Notably, the addition of LCA does not significantly impact the ζ potential, a feature that was critical in the molecular design. Further, all NPs have low PDIs, indicating uniform NP size with no evidence of NP aggregation. This attribute is an important feature to ascertain oxLDL uptake competitive inhibition and prolonged storage stability. 

### 3.3. AScM NPs Inhibit oxLDL Uptake in Human Macrophages

**Figure 4:**
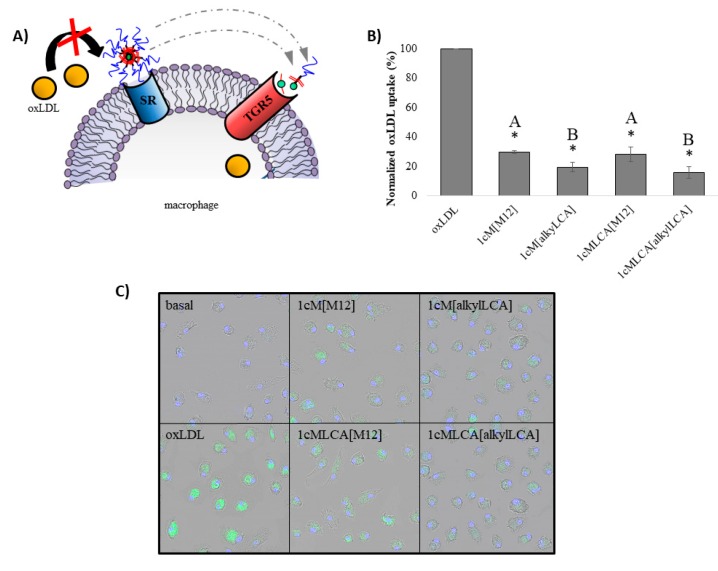
AScM NPs inhibit oxLDL uptake in macrophages (schematic, **A**). All NPs lower oxLDL uptake in hMDMs compared to basal controls as measured via flow cytometry and indicated with (*) (**B**) Core chemistry has a significant impact on oxLDL uptake inhibition levels, but shell chemistry does not significantly alter efficacy as indicated by letters. Bars with different letters represent statistical differences. Confocal microscopy images of oxLDL uptake by hMDMS 24 h after treatment with AScM NPs cell nuclei (DAPI, blue), and DiO labeled oxLDL (DiO, green) 40× magnification (**C**).

To measure oxLDL uptake inhibition, hMDMs were simultaneously treated with AScM NPs and oxLDL or oxLDL alone (control), and internalization was quantified using flow cytometry and visualized by confocal microscopy (Figure 4). Cells were treated at 1 × 10^−5^ M concentration of the AScMs, as we have previously demonstrated in vitro and in vivo cytocompatibility and high AScM bioactivity at this concentration [17,19]. Additionally, all AScM NPs described herein have cytocompatibility >80% (*data not shown*). Under all AScM NP treatments, oxLDL uptake was reduced to <30% of controls (Figure 4B). These data demonstrate that both shell materials are highly efficacious at reducing oxLDL internalization, including NPs with LCA-conjugation. We have previously demonstrated that NPs based on polystyrene and poly(lactic acid) are inactive [17,20]. As such, control experiments utilizing NPs with experimental shells and a polystyrene core, as well as NPs with a polystyrene-PEG shell and experimental cores were carried out. These experiments indicate that both experimental components are, in fact, active. This observation supports our hypothesis that the NPs competitively inhibit SRs, including those with LCA conjugation, while they retain potent oxLDL uptake inhibition properties. The NPs with LCA’s free carboxylate retain a negative ζ potential and have sizes amenable to endocytosis, which would give rise to potent oxLDL competitive inhibition. These observations are in agreement with previous findings for other formulations, in which NPs with weakly negative ζ potentials exhibited poor inhibition of oxLDL uptake compared to NPs with higher magnitudes of negative ζ potential [20]. Therefore, with the criteria of a negative NP ζ potential and NP sizes below 200 nm, it is plausible that an array of naturally active ligands conjugated to 1cM would elicit desired biological effects. 

While all shells were found to be active, these data further illustrate that shell chemistries that contain the 1cM base structure do not adversely influence the magnitude of oxLDL uptake inhibition (Figure 4B). Both the 1cM and 1cMLCA shells exhibit comparable degrees of oxLDL uptake inhibition. Interestingly, the NP core material influences the degree of oxLDL uptake, with alkylLCA cores exhibiting significantly more oxLDL uptake inhibition than M12 cores (Figure 4B). The stronger influence of the core material on the NP anti-atherogenic potential is consistent with previously reported data using mixed PS and M12 cores [20]. As such, we hypothesize that oxLDL uptake is not only lowered by competitive interactions with SRs. Rather, it is likely that surface molecules from the NPs’ shells exist in an equilibrium with the unimeric components. This equilibrium may be mediated by extracellular serum disruption or intracellular mechanisms, which leads to interactions between the core materials and biological targets. For example, 1cMLCA[alkylLCA] NPs may partly dissociate to free their respective amphiphilic unimers, thereby allowing them to interact with proximal cellular receptors, such as TGR5, to elicit an enhanced reduction in oxLDL uptake (Figure 4A). We have demonstrated the biodegradation of AScM ester functionalities by serum esterases in previous work, which presumably would lead to high local concentrations of free LCA upon cleavage of alkylLCA [34]. The high local concentration of LCA is hypothesized to be partially responsible for increased oxLDL reduction for NPs with alkyl LCA cores, as TGR5 activation has a demonstrated effect on reducing oxLDL uptake [22]. 

### 3.4. AScM NP Composition Markedly Impacts Inflammatory Gene Transcription

Gene expression was used to evaluate the LCA-based NPs potential to decrease inflammation in macrophages. LCA-conjugates were strategically designed to interact with membrane bound receptors, including TGR5, to mitigate the inflammatory response caused by the atherosclerotic cascade, as well as from NP administration. TGR5 is a transmembrane protein expressed in macrophages that has a demonstrated role in the reduction of several inflammatory cytokines in the atherosclerotic cascade. Of interest, decreases in the transcription and expression of IL-1β, IL-6, and IL-8 have been demonstrated upon treatment with bile acids or synthetic TGR5 agonists [25,35,36]. These genes are also upregulated in human macrophages following treatment with 1cM[M12] NPs, which limits this NP formulation’s clinical applicability [18]. As such, qRT-PCR was utilized to evaluate the potential influence of NP composition on the inflammatory cytokine profile in human macrophages, including those genes known to mitigate and exacerbate the disease state.

**Figure 5:**
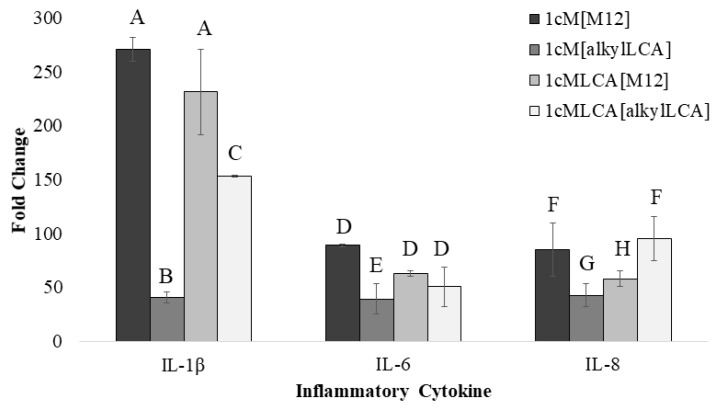
Fold change in mRNA levels of inflammatory cytokines and scavenger receptors (SRs) in hMDMs as determined by rt-PCR 24 h after treatment with AScM NPs and oxLDL as compared to untreated (basal) controls. Transcription levels of three cytokines significantly affected by NP treatment. Different letters indicate statistical significance, whereas identical letters indicate no statistical difference.

The simultaneous treatment of macrophages with AScM NPs and oxLDL resulted in changes in mRNA expression of inflammatory cytokines mediated by TGR5 and SRs involved in oxLDL uptake (Figure 5). Findings are consistent with previous studies, indicating that NPs containing an M12 core significantly upregulate mRNA expression of the pro-atherogenic cytokine IL-1β [18]. IL-1β is a pro-inflammatory cytokine secreted by activated monocytes and macrophages and is implicated in both the early and late stages of atherosclerosis [37]. In the early stages of atherosclerosis, IL-1β increases adhesion molecule expression in endothelial cell membranes, while in later stages, it facilitates fibrous cap destabilization at plaque sites in vivo, ultimately leading to plaque rupture [37,38]. As such, the increase in IL-1β transcription induced by 1cM[M12] NPs is undesirable, and treatment with these NPs would exacerbate atherogenesis. In this work, the increase in IL-1β observed after 1cM-based NP treatment was significantly lower with alkylLCA incorporation into the NPs cores as compared to those with M12 cores (Figure 5). This effect was also observed for NPs with 1cMLCA shells. These data demonstrate that core modification can mitigate the limitations of previous AScM NP formulations. We hypothesized that LCA-incorporation into the shell would further downregulate IL-1β expression due to TGR5 activation. Interestingly, it was observed that the attenuation of IL-1β transcription with 1cMLCA shells was not as prominent, suggesting that inflammation resulting from NP treatment is multifactorial, and more largely influenced by the hydrophobe component of NPs. As 1cMLCA-based NPs exhibit a minimal response to core identity, it is possible the overall NP hydrophobicity also contributes to IL-1β expression. 

1cM[M12] also results in upregulation of the pro-atherogenic cytokines IL-6 and IL-8 in macrophages, limiting its further development as a comprehensive therapeutic against athero-inflammation [18]. IL-6 has been implicated in recruitment of activated monocytes and their subsequent differentiation into macrophages, and an increase in IL-8 upregulates endothelial expression of adhesive molecules that promote activated monocyte/macrophage attachment [38]. Both of these steps occur in the early stages of atherosclerosis, and preventing or reducing their occurrence is crucial to halting the cascade’s initiation and perpetuation. As such, the reduction in the secretion of these cytokines is needed to increase the therapeutic potential of AScM NPs. Expression of IL-6 and IL-8 with AScM NP treatment reveal similar trends to those observed for IL-1β, with a decrease in transcription when macrophages are treated with 1cM-based NP formulations with alkylLCA cores. Interestingly, no notable trend is observed for expression of either IL-6 or IL-8 in macrophages treated with 1cMLCA NPs, further supporting the hypothesis that the level of overall AScM NP hydrophobicity is critical in mitigating the inflammatory response in macrophages treated with NPs. 

Additional inflammatory cytokines were evaluated including IL-10 and TNFα, as well as two SRs responsible for oxLDL internalization, CD36 and SRA. In all cases, NP formulations had minimal influences on these genes’ mRNA expression. These data suggest that the AScM NPs’ molecular mechanism leading to inflammation reduction may occur via an alternative mechanism to mRNA control of protein expression, consistent with previous work demonstrating transient effects on macrophage cytokines [11]. Nonetheless, 1cM[alkylLCA] was identified as a lead NP formulation, as it demonstrates a large reduction in several inflammatory cytokines, namely IL-1β, IL-6, and IL-8, induced via atherosclerosis and administration of previous AScM NP formulations. 

## 4. Conclusions

LCA was modified and effectively incorporated into NPs to establish a new generation of NPs for the treatment of inflammation in atherosclerosis. The NPs were comprised of an LCA-containing shell, where the bile acid was conjugated to AScMs known to limit uncontrolled oxLDL uptake in human macrophages, and an LCA-containing core, where LCA was hydrophobically modified to serve as a NP nucleation point [10]. The appropriate size for cellular uptake and net negative charge allow the NPs to act similarly to oxLDL and inhibit its uptake via competition with oxLDL in SR binding sites. 

NPs with alkylLCA cores lowered the atherogenic potential of macrophages by significantly decreasing oxLDL uptake compared to NPs with M12 cores. However, the shell composition did not influence oxLDL uptake inhibition, indicating that the core exerts an active effect over the efficacy. It is likely that AScM NPs exist in equilibrium with monomeric components, and serum proteases partly degrade the hydrophobic cores. We hypothesize that this equilibrium would result in interactions between the natural ligand, LCA, with TGR5, resulting in receptor activation and a decrease in oxLDL uptake. Both the core and shell components did, however, impact the gene transcription profile in macrophages treated with AScM NPs. Treatment with 1cM[alkylLCA] NPs resulted in the lowest mRNA expression levels of inflammatory cytokines in macrophages treated with AScM NPs to date. 

Together, this information illustrates that modifying AScM NP composition can have a pronounced impact on the inflammatory profile of human macrophage populations. The inflammatory cytokines evaluated in this study that were transcriptionally downregulated in the presence of the 1cM[alkylLCA] are, in part, regulated by TGR5 activation. It is therefore possible that this NP formulation activates TGR5, leading to a decrease in inflammation. As it appears that the overall hydrophobicity influences the inflammatory response, this formulation also appears to have the optimal balance of hydrophobicity imparted by alkyl chains and LCA moieties to not exacerbate inflammation while simultaneously reducing inflammatory cytokine expression involved in atherosclerosis. 

These findings illustrate that our current class of AScM nanotherapeutics can be modified using complementary ligands to further fine-tune desired biological outcomes without compromising the inherent anti-lipogenic profile of previous AScM NPs. As both a reduction in oxLDL uptake and inflammation are critical to ameliorate macrophage atherogenic potential, the results of these experiments demonstrate improvement upon previous AScM generations with respect to inflammatory cytokine expression and provide insight to develop future AScM NPs that exhibit desirable outcomes by influencing biological targets.

## Figures and Tables

**Table 1:** Physicochemical properties of AScM NPs indicating particle size, polydispersity index (PDI) and ζ potential determined by dynamic light scattering measurements. Formulation notation is denoted as Shell[Core], and statistical differences are indicated by different symbols.

Formulation	Hydrodynamic Diameter (nm)	PDI	ζ Potential (mV)
1cM[M12]	213 + 4 *	0.11	−30
1cM[alkylLCA]	206 + 16 *	0.09	−31
1cMLCA[M12]	176 + 14 ǂ	0.16	−33
1cMLCA[alkylLCA]	173 + 2 ǂ	0.11	−31
